# Xq27.1 palindrome mediated interchromosomal insertion likely causes familial congenital bilateral laryngeal abductor paralysis (Plott syndrome)

**DOI:** 10.1038/s10038-022-01018-z

**Published:** 2022-01-31

**Authors:** Felix Boschann, Daniel Acero Moreno, Martin A. Mensah, Henrike L. Sczakiel, Karolina Skipalova, Manuel Holtgrewe, Stefan Mundlos, Björn Fischer-Zirnsak

**Affiliations:** 1 Charité – Universitätsmedizin Berlin, corporate member of Freie Universität Berlin and Humboldt-Universität zu Berlin, Institute of Medical Genetics and Human Genetics, Berlin, Germany; 2 Division of Pediatric Critical Care Medicine, Kliniken der Stadt Köln gGmbH, Cologne, Germany; 3 Clinician Scientist Program, Berlin Institute of Health at Charité – Universitätsmedizin Berlin, BIH Academy, Berlin, Germany; 4 Core Facility Bioinformatics, Berlin Institute of Health Charité – Universitätsmedizin Berlin, Berlin, Germany; 5 RG Development & Disease, Max Planck Institute for Molecular Genetics, Berlin, Germany

**Keywords:** Genetics research, Genetic translocation

## Abstract

Bilateral laryngeal abductor paralysis is a rare entity and the second most common cause of stridor in newborns. So far, no conclusive genetic or chromosomal aberration has been reported for X-linked isolated bilateral vocal cord paralysis, also referred to as Plott syndrome. Via whole genome sequencing (WGS), we identified a complex interchromosomal insertion in a large family with seven affected males. The 404 kb inserted fragment originates from chromosome 10q21.3, contains no genes and is inserted inversionally into the intergenic chromosomal region Xq27.1, 82 kb centromeric to the nearest gene SOX3. The patterns found at the breakpoint junctions resemble typical characteristics that arise in replication-based mechanisms with long-distance template switching. Non protein-coding insertions into the same genomic region have been described to result in different phenotypes, indicating that the phenotypic outcome likely depends on the introduction of regulatory elements. In conclusion, our data adds Plott syndrome as another entity, likely caused by the insertion of non-coding DNA into the intergenic chromosomal region Xq27.1. In this regard, we demonstrate the importance of WGS as a powerful diagnostic test in unsolved genetic diseases, as this genomic rearrangement has not been detected by current first-line diagnostic tests, i.e., exome sequencing and chromosomal microarray analysis.

## Introduction

Vocal cord paralysis is the second most common cause of stridor in newborns, which can lead to respiratory distress and often requires intubation or tracheostomy [1, 2]. Frequent causes of idiopathic and acquired cases are primary neurological defects, birth trauma, infections or surgical procedures [3]. Familial cases of bilateral congenital vocal cord paralysis are rare and mostly associated with other syndromic malformations [4]. X-linked isolated bilateral vocal cord paralysis, also referred to as Plott syndrome (MIM: 308850) has been described in three different families [5–7]. Since the paralysis can improve over time, it was assumed that the cause was the immaturity of the chemoreceptive pathway between the nucleus ambiguus, the carotid body and the posterior cricoarytenoid muscle [4]. So far, no conclusive genetic or chromosomal aberration has been reported [5–7]. Likewise, up to 60% of assumed monogenetic diseases remain unsolved using current first-tier diagnostic tests (e.g., chromosomal microarray analysis (CMA) and exome sequencing (ES)) [8, 9]. A shortcoming of these methods is the inaccurate detection of structural variants (SVs, i.e., DNA rearrangements comprising more than 50 nucleotides). On average more than 25k SVs are present in every human genome and thereby represent the largest source of genomic diversity [10–13]. Rare, disease-causing SVs can vary widely in size and include copy number alterations (CNVs: deletions and duplications) and copy-number neutral changes such as inversions and translocations [14]. The “upstream” mechanisms leading to the formation of SVs can be categorized into recombination-based (e.g., non-allelic homologous recombination, NAHR) and replication-based mechanisms (RBM) [15]. The “downstream” mechanisms leading to phenotypes are manifold [16, 17]. Aside alterations of gene dosage or disruptions of coding sequence, SVs affecting the non-coding part of the genome can alter 3D chromatin architecture, which can result in misregulated gene expression [18, 19].

Herein we report a new family with Plott syndrome and the identification of a novel interchromosomal insertion via whole genome sequencing (WGS) as the likely cause of X-linked isolated congenital bilateral vocal cord paralysis.

## Material and methods

### Ethics statement

Consent of each participating individual or their legal guardian was obtained for all clinical and molecular studies of this report and for the publication of clinical photographs. All studies and investigations were performed according to the declaration of Helsinki principles of medical research involving human subjects.

### Molecular genetic analysis

#### Short-reads WGS

Genomic DNA from affected individual V-3 and his unaffected brother (V-2) was isolated from peripheral blood for subsequent genome sequencing on an Illumina platform using the TruSeq DNA PCR-free protocol (Macrogen). Reads were aligned to human genome build GRCh37/hg19 using BWA-MEM 0.7.17 (arXiv:1303.3997v2). Structural variants were called using Delly v0.8.1 (PMID: 22962449) [20] and analyzed according to an in-house standard operating procedure using the VarFish platform [21]. The BAM files were manually inspected for variants of interest in the Integrative Genome Viewer (IGV, http://software.broadinstitute.org/software/igv/). Exact determination of breakpoint junction at nucleotide resolution was possible via alignment of split reads and further examination via the UCSC blat tool (https://genome.ucsc.edu/cgi-bin/hgBlat).

#### Breakpoint junction PCR

Genomic DNA was amplified using primer pairs spanning both breakpoint junctions (primer sequences are listed in Supplementary Table 1). Fragments were sequenced directly after enzymatic purification by shrimp alkaline phosphatase and exonuclease 1 using BigDye^®^ Terminator v3.1 (Applied Biosystems) and run on ABI 3730 DNA Analyzer (Applied Biosystems).

#### Quantitative real-time polymerase chain reaction (qPCR)

We performed qPCR using genomic DNA of the index subject and family members to confirm the duplication of the 10q21.3 region and show segregation with the phenotype. The relative copy number (RCN) of the target sequence was measured using EVAgreen (Solis BioDyne) and quantified using the comparative ΔΔCt method on a QuantStudio 3 Real-Time-PCR System (ThermoFisher Scientific) in comparison to Albumin. The primer sequences are given in Supplementary Table 1.

#### Chromosomal microarray

CMA for individual V-3 was carried out using a whole-genome 1 M oligonucleotide array (Agilent; Santa Clara, CA). Data were analyzed as previously reported [22].

#### FISH

Fluorescent in situ hybridization (FISH) analyses were performed on metaphase spreads prepared from 72 h PHA stimulated peripheral blood lymphocyte culture from the index patient (V-3) according to standard procedures following respective manufacturer’s protocol. The BAC clone RP11-176H12 (Empire Genomics, Buffalo, NY) in color *aqua* was selected to cover the duplicated region of 10q21.3. Vysis CEP 10 SpectrumOrange Probe (Abbott) and Vysis CEP X (DXZ1) SpectrumGreen Probe (Abbott) were used to label centromeres of chromosome 10 and chromosome X.

## Results

### Clinical report

The affected boy (V-3) is the second child of non-consanguineous parents. He was born at 40 weeks of gestation with the following birth measurements: weight 3200 g (P42, −0.96 SD), length 50 cm (P54, −1.09 SD), head circumference 35 cm (P65, −0.46 SD). There were no congenital malformations or comorbidities. Shortly after birth, stridor was noticed and due to respiratory distress, mechanical ventilation was necessary. Multiple extubation attempts were frustrated by recurrence of stridor. A tracheobronchoscopy showed significant ulceration of the mucosa at the level of the arytenoid cartilage on the left, corresponding to damage due to long-term intubation. Submucosal injection of Betamethasone in the affected area was applied. The subglottic space was unremarkable. After a new extubation attempt, the child showed recurrence of stridor with progressive respiratory failure necessitating reintubation. The follow-up tracheobronchoscopy showed vocal folds in an intermediate position. Since no vocal fold abduction during inspiration was achieved, a congenital bilateral vocal cord paresis was suspected.

An MRI of the neck and brain showed no abnormalities. A tracheostomy was performed without complications and the patient was subsequently discharged. A tracheoscopy at the age of 15 months showed a clear mobility of the right vocal fold and a still uncoordinated lateralization and fasciculation on the left side. At the most recent physical examination at the age of 24 month, he is still fitted with a tracheostoma. His psychomotor development is regular. CMA and ES did not show conclusive findings. Family history revealed that seven male neonates had acute inspiratory stridor and died within the first days of life due to respiratory distress. No autopsy or genetic testing had been performed on any of them.

**Fig. 1:**
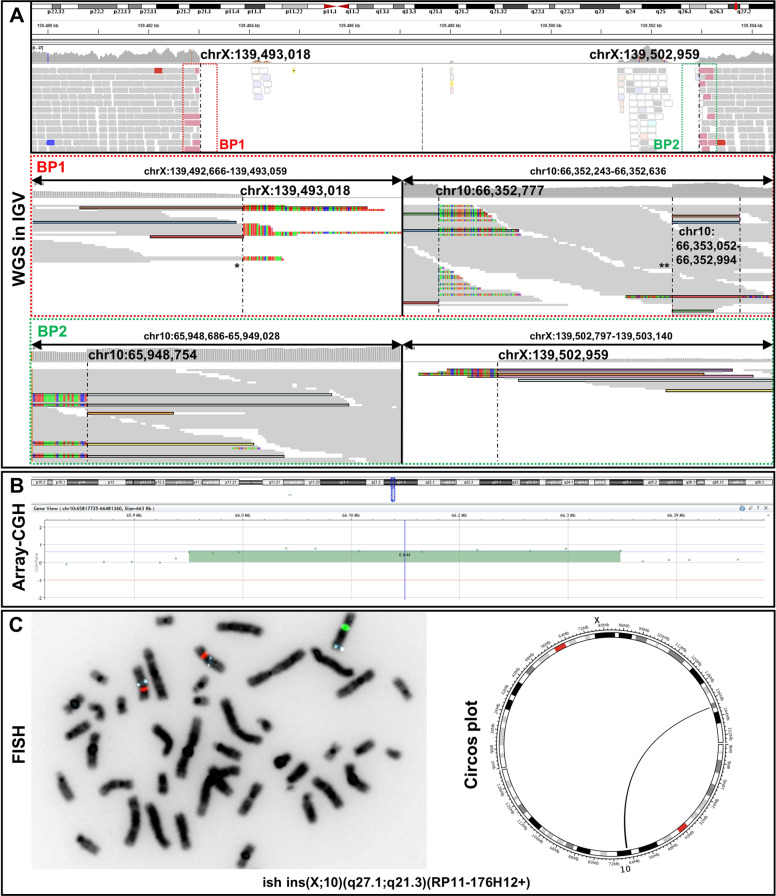
Molecular genetic findings. **A** Split-reads and discordant pairs retrieved from WGS and visualized on IGV are shown in different colors matching for their pairs. Non-split-reads that map to the genome of reference are shown in gray. Dashed vertical lines represent the breakpoints. WGS identified a ~10 kb spanning deletion in the intergenic region of Xq27.1 [NC_000023.10:g.139493018_139502959]. The proximal breakpoint junction (BP1) comprises two joint points (indicated by asterisks). The first joint-point * connects chromosome X to chromosome 10q21.3. After the insertion of 59 bp another break occurs (second joint point **). Subsequently, the sequence of the large 404 kb fragment continues, which is located 219 bp centromeric. The distal breakpoint junction (BP2) connects the large duplicated fragment of chromosome 10q21.3 back to chromosome X. The junctions are shown at the basepair level in Supplementary Fig. 1. **B** Array CGH shows a 404 kb spanning region of chromosome 10q21.3 to be duplicated. **C** FISH signals on metaphase chromosomes showing the interchromosomal insertion. BAC probes are RP11-176H12 (10q21.3 - aqua), Vysis CEP 10 SpectrumOrange Probe (10p11.1-q11.1,- orange) and Vysis CEP X (DXZ1) SpectrumGreen Probe (Xp11.1-q211.1 - green). ISCN: ish der(X)ins(X;10)(q27.1;q21.3)(RP11-176H12+). Schematic representation of the interchromosomal insertion as circos plot retrieved from WGS data.

### Molecular genetic findings

More than 115 Gb of sequences were generated for each individual (V-2 and V-3). More than 97% of sequenced bases had a Phred quality score of over 20 and >92.6% of over 30. WGS data evaluation revealed no convincing coding variant, however, identified a ~10 kb spanning deletion in an intergenic region on chromosome Xq27.1 [NC_000023.10:g.139493018_139502959] (Fig. 1A) Split reads were visible at both breakpoints, with the non-aligned reads each mapping to two genomic regions on chromosome 10q21.3 [NC_000010.10:g.A66353052 and NC_000010.10:g.65948754] (Fig. 1A). HGVS: NC_000023.10:g.139493018_139502959ins [NC_000010.10:g.66353052_65948754inv]. These genomic locations represent the boundaries of a 404 kb large fragment. Depth of coverage data and the previously performed CMA confirmed that this region of chromosome 10q21.3 is duplicated (Fig. 1B). FISH analysis showed the presence of an additional aqua signal (representing the 10q21.3 region) on chromosome X (green), thereby confirming the interchromosomal insertion event (Fig. 1C). ISCN nomenclature: ish der(X)ins(X;10)(q27.1;q21.3)(RP11-176H12+).

**Fig. 2:**
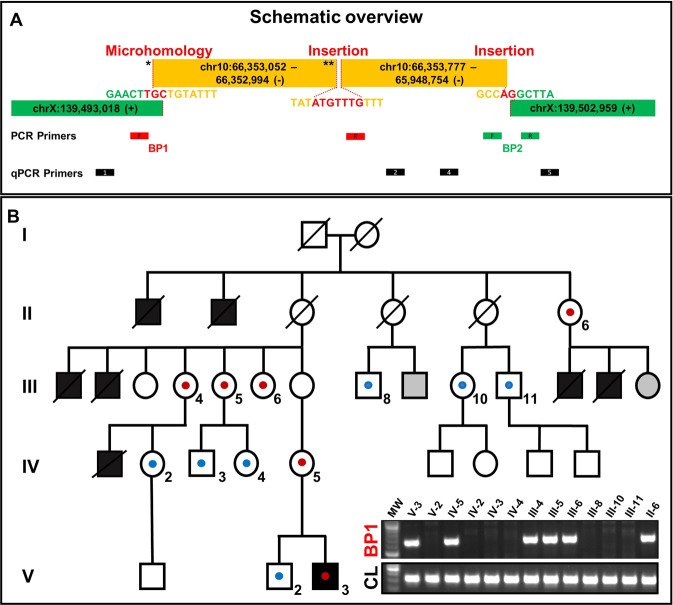
Schemativ overview of the complex chromosomal rearrangement and Segregation analysis. **A** The patterns found at the two breakpoint junctions (i.e. microhomology, small insertions, large template insertion, deletion at insertion site) resemble typical characteristics that arise due to replication-based repair mechanisms like MMBIR/FoSTes. The proximal breakpoint junction (BP1) comprises two joint points (indicated by asterisks). Location of primers for break point spanning PCR and qPCR. **B** Pedigree showing our proband (V-3) and his five-generation family with seven affected male neonates. Schematic view of the co-segregation analysis: blue dots indicate presence of the wild type chromosome X, red dots show the presence of the derivative chromosome X. PCR amplification of the proximal breakpoint junction (BP1) (exemplary). CL control locus PCR, gray filled = not consented for testing.

For accurate breakpoint determination, we performed breakpoint-spanning PCRs. For the proximal breakpoint junction (BP1), the exact location of the end sequence from chromosome X and start position of the 10q21.3 inserted sequence (joint point 1 *) could not be unambiguously defined due to a 3-bp overlap/microhomology (“TGC”) in the sequence (Fig. 2A and Supplementary Fig. 1). After the insertion of 59 bp [NC_000010.10:66352994-66353052], a new break occurs (joint point 2 **). The following sequence also originates from chromosome 10q21.3 and is located 217 bp centromeric (NC_000010.10:66352777). Between these parts 7 bp of unknown origin are inserted. The distal breakpoint junction (BP2) is formed by the end of the large duplicated fragment (NC_000010.10:65948754) and chromosome X (NC_000023.10:139502959). At this junction we found a 2 bp insertion (“AG”) (Fig. 2A and Supplementary Fig. 1).

Segregation analysis was performed using breakpoint specific PCRs and qPCR. By this approach we confirmed the derivative X-chromosome in the affected individual V-3. In addition, we found this genomic rearrangement in the female individuals IV-5, III-4, III-5, III-6 and II-6 (Fig. 2B, Supplementary Fig. 2).

## Discussion

Bilateral vocal cord paralysis is a rare entity with an incidence less than 1/100.000 [1]. In 1964 Plott and colleagues first described bilateral familial laryngeal abductor paralysis in four male siblings and assumed X-linked inheritance [2]. Since then, two other families with suspected Plott syndrome (MIM: 308850) have been reported, but no genetic tests had been performed [6, 7]. Furthermore, it has not been elucidated whether the laryngeal abductor paralysis is caused by a central lesion at the level of the ventral division of the nucleus ambiguus or by a peripheral myogenic process. Via WGS, we identified a complex interchromosomal insertion as the likely cause of X-linked congenital bilateral laryngeal paralysis, i.e., Plott syndrome, in an affected boy with a conspicuous family history. Testing of the other six affected boys was not possible because they died several decades ago. However, segregation analysis revealed that each mother of an affected child carries the genomic rearrangement, whereas it was not detectable in any unaffected male family member.

Interchromosomal insertions are complex genomic rearrangements (CGR), characterized by chromosomal segments inserted into an interstitial region of non-homologous chromosome generated by more than three DNA breakage and joining events [23]. Approximately 2% of non-recurrent copy number gains are caused by inter- or intrachromosomal insertions [24]. The initial trigger of the herein reported interchromosomal insertion is most likely the human specific AT-rich palindrome located in the 485 kb spanning intergenic chromosomal region Xq27.1, 82 kb centromeric to the nearest gene *SOX3* [25]. Distinct phenotypes, such as congenital hypertrichosis, congenital ptosis, Charcot–Marie–Tooth neuropathy and sex reversal have been reported with different inter- and one intrachromosomal insertions into the above-mentioned non-coding region [25–31].

Since palindrome sequences can form hairpin loops, they are prone to DNA double-strand breaks and thus pose a risk for the initiation of chromosomal rearrangements [32, 33]. As in our case (distal breakpoint junction BP2), at least one breakpoint of the other reported cases was located near the center of this palindromic sequence [28]. The reestablishment of a broken replication fork depends on stretches of microhomologies which are used by error prone DNA polymerases for priming synthesis on a template strand, a mechanism called Microhomology-Mediated Break-Induced Replication (MMBIR) [33, 34]. Such replication-based repair mechanisms are assumed to have the largest contribution in the formation of nonrecurrent structural rearrangements [33, 35]. These “upstream” mechanisms can often be identified by specific signatures at the breakpoint sequence. This is a major advantage of genome sequencing, since, in contrast to CMA or ES, accurate nucleotide resolution of breakpoint junctions is possible. In our case, the proximal breakpoint junction (BP1) comprises two joint points. At the first joint point we found a microhomology of 3-bp. After the insertion of 59 bp, another break occurs, followed by the insertion of 7 bp of unclear origin. Subsequently, the sequence of the large 404 kb fragment continues, which is located 219 bp centromeric. This discontinuous transition indicates that the polymerase has reduced processivity and that a stable replisome is established only after iterative template switches. The distal breakpoint displays features of a simple breakpoint junction. Taken together the patterns found at the two breakpoint junctions (i.e., microhomologies, small insertions, large template insertion, deletion at insertion site) resemble typical characteristics that arise in replication-based mechanisms with long-distance template switching (i.e., MMBIR/and Fork Stalling and Template switching (FoSTes)) [33]. Notably, both ends of the sequences that form the proximal junction are located within LINE1-Elements, demonstrating that repetitive elements further stimulate the formation of complex insertions by providing sequences with microhomologies.

The “downstream” mechanisms of genomic rearrangements leading to phenotypic consequences can be caused by various mechanisms. In addition to direct disruption of the coding region of genes, insertions affecting intergenic regions can modify higher-order chromatin organization such as topologically associating domains (TADs) [19].

In this case, the inserted 404 kb large fragment contains no genes. Other cases featuring non-coding insertions at this position resulted in varying other phenotype, indicating that the phenotypic outcome might result from “enhancer-adoption” (i.e., the introduction of tissue specific enhancers resulting in ectopic expression of *SOX3*). Unfortunately, due to absent *SOX3* expression in tissues accessible from patients, we were unable to perform precise gene expression analyses, a problem that has already been addressed by Si and colleagues [26]. SOX3, together with SOX1 and SOX2, forms the SOXB1 subgroup and is an early marker of vertebrate neurogenesis [36]. *SOX3* is mainly expressed in telencephalic neuronal precursor cells (NPC) throughout neuroaxis and binds to neuronal genes, thereby priming them for subsequent activation by SOX11 in differentiating neurons. *SOX3* regulation and expression appears to be tissue specific. During neuronal differentiation, *SOX3* is generally downregulated but during hypothalamic development *SOX3* remains expressed in a subset of differentiated hypothalamic cells in the adult brain [37, 38]. Notably, *SOX3* is crucial for early segmentation of pharyngeal pouches and proper neural crest cell migration into pharyngeal arches and thereby for cranial nerve and epibranchial placode formation [39, 40]. Thus, *SOX3* might also be an important regulator for the chemoreceptive pathway between the nucleus ambiguus, the carotid body and the posterior cricoarytenoid muscle, the perturbation of which is thought to be the cause of Plott syndrome [4]. We therefore hypothesize that altered regulation and ectopic expression of *SOX3* caused by the identified complex interchromosomal insertion is the cause of X-linked congenital bilateral laryngeal abductor paralysis (Plott syndrome).

Further studies investigating the effect on *SOX3* expression in neuronal tissue (iPS-induced) and molecular characterization of additional individuals with Plott syndrome would strengthen our assumption.

This study emphasizes that distinct diseases can result from insertions into the same non-coding genomic region and that the phenotypic outcome likely depends on the introduction of regulatory elements. In this regard, we demonstrate the importance of WGS as a powerful diagnostic test in unsolved rare cases, even though detection of structural variations with short-read technology has its imitations [5, 32]. Even if accurate detection of complex structural rearrangements and their breakpoint junctions by long read sequencing approaches becomes feasible, the interpretation of non-coding genomic rearrangements remains challenging [41, 42]. This requires the integration of multimodal layered biological data (gene expression, epigenetics, 3D structure) to functionally interpret their effect within multiple molecular contexts.

## Supplementary information

Supplement

## Data Availability

All data relevant to the study are included in the article or the Supplementary Information. For further information, contact the corresponding author.
