# Supplementary material for: Xq27.1 palindrome mediated interchromosomal insertion likely causes familial congenital bilateral laryngeal abductor paralysis (Plott syndrome)
Source: J Hum Genet. 2022 Jan 31;67(7):405–10. doi: 10.1038/s10038-022-01018-z (PMC9233990; doi:10.1038/s10038-022-01018-z)
Supplement: Supplementary file 1 — Supplement [file 10038_2022_1018_MOESM1_ESM.pdf]

Supplementary Figure 1

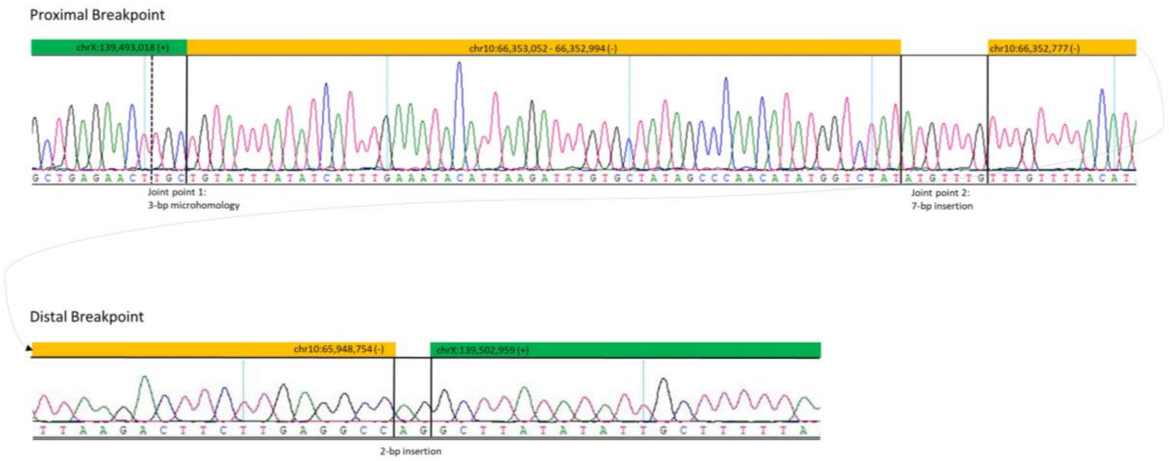

Sequence analysis of the proximal and distal breakpoint junctions at base-pair resolution. Between the green marked reference sequence on chromosome X, the inversely inserted duplicated sequence from chromosome 10 is shown in yellow.

Supplementary Figure 2

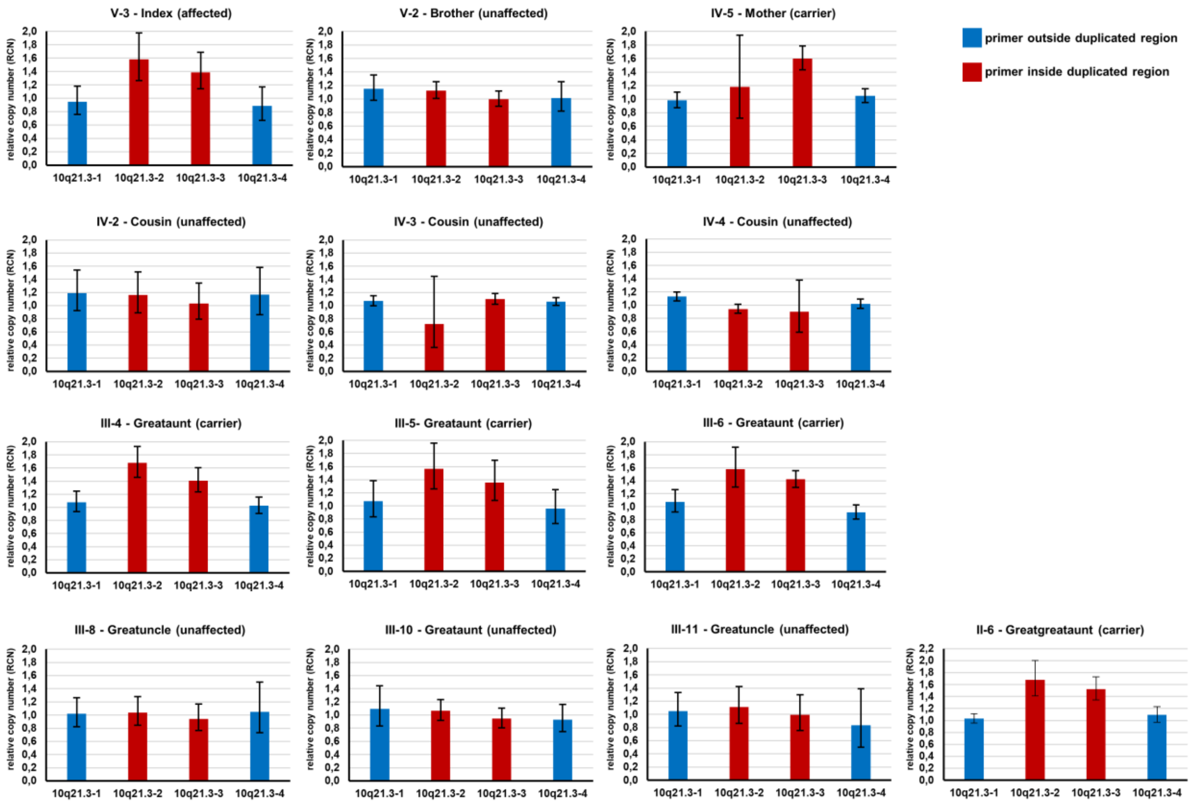

Validation of the duplication and its segregation by qPCR. For all DNA samples available from the family two primer pairs adjacent (blue) and inside (red) the duplicated region respectively were used to investigate the relative copy number for these regions. By this approach, we confirmed the presence of the duplication in the index patient (V-3) and found the female individuals IV-5, III-4, III-5, III-6 and II-6 to be obligate carriers. RCN, relative copy numbers, Error bars indicate SD.

Supplementary Table 1

breakpoint PCR		
	Forward (5'-3')	Reverse (5'-3')
BP1	GGAGGTGGCAATTTCAAGAG	CCAAGGTAGGCTTCAAGACAAG
BP2	AGTGGGTAGAAGCCGGAGA	AGTGGCTTTGTTTCCACCAG
qPCR		
	Forward (5'-3')	Reverse (5'-3')
10q21.3-1	GGCCTCTACTTGTGAGGTGC	GTAGGCATATCTCCGCCACC
10q21.3-2	ACAGGGCTAGGCAGGTAAGA	TCTAGCCAGGGCCTGTACTT
10q21.3-4	GCCTTGGCTGTGGGTAGTAT	AAGGTCTCTCCACTCAGGC
10q21.3-5	CCCAAGGCTTATCTTTGGAGGA	GTTGAGCAAGGAGCTTTCTTCTG
